# Case Report: Compound heterozygous nonsense mutations in *TRMT10A *are associated with microcephaly, delayed development, and periventricular white matter hyperintensities

**DOI:** 10.12688/f1000research.7106.1

**Published:** 2015-09-28

**Authors:** Mohan Narayanan, Keri Ramsey, Theresa Grebe, Isabelle Schrauwen, Szabolcs Szelinger, Matthew Huentelman, David Craig, Vinodh Narayanan, C4RCD Research Group

**Affiliations:** 1 Arizona Pediatric Neurology & Neurogenetics Associates, Phoenix, AZ, USA; 2 Barrow Neurological Institute, Phoenix, AZ, USA; 3 Center for Rare Childhood Disorders, Translational Genomics Research Institute, Phoenix, AZ, USA; 4 Neurogenomics Division, Translational Genomics Research Institute, Phoenix, AZ, USA; 5 Department of Genetics, Phoenix Children’s Hospital, Phoenix, AZ, USA

**Keywords:** Primary microcephaly, Delayed development, Periventricular leukomalacia, White matter hyperintensities, Diabetes mellitus, TRMT10A, tRNA methyltransferase

## Abstract

Microcephaly is a fairly common feature observed in children with delayed development, defined as head circumference less than 2 standard deviations below the mean for age and gender. It may be the result of an acquired insult to the brain, such prenatal or perinatal brain injury (congenital infection or hypoxic ischemic encephalopathy), or be a part of a genetic syndrome. There are over 1000 conditions listed in OMIM (Online Mendelian Inheritance in Man) where microcephaly is a key finding; many of these are associated with specific somatic features and non-CNS anomalies. The term primary microcephaly is used when microcephaly and delayed development are the primary features, and they are not part of another recognized syndrome.

In this case report, we present the clinical features of siblings (brother and sister) with primary microcephaly and delayed development, and subtle dysmorphic features. Both children had brain MRI studies that showed periventricular and subcortical T2/FLAIR hyperintensities, without signs of white matter volume loss, and no parenchymal calcifications by CT scan. The family was enrolled in a research study for whole exome sequencing of probands and parents. Analysis of variants determined that the children were compound heterozygotes for nonsense mutations, c.277C>T (p.Arg93*) and c.397C>T (p.Arg133*), in the *TRMT10A* gene. Mutations in this gene have only recently been reported in children with microcephaly and early onset diabetes mellitus.

Our report adds to current knowledge of *TRMT10A* related neurodevelopmental disorders and demonstrates imaging findings suggestive of delayed or abnormal myelination of the white matter in this disorder. Accurate diagnosis through genomic testing, as in the children described here, allows for early detection and management of medical complications, such as diabetes mellitus.

## Clinical summary

We present case histories of siblings (a sister and brother) born to healthy non-consanguineous parents, with microcephaly and delayed development.

### Patient 1

The first patient is a 12 year old girl who was born at 39 weeks gestation weighing 4 lb 10 oz, without perinatal difficulties. She was healthy as an infant, except for recurrent ear infections, which were treated with tympanostomy tubes. Hypotonia was noted during infancy, but she reached her early motor milestones, including crawling and walking, at the normal ages. Speech delay and behavioral problems were noted by 4 years of age. There was no regression. Ophthalmological evaluation was normal. Physical exam at age 4.5 years showed microcephaly, low anterior hair line, deep set eyes with mild hypotelorism, shortened forehead, and her neurological exam was normal. Head circumference was 3.5 SD below the mean for age in girls. These features have remained constant at subsequent examinations.

**Figure 1.**
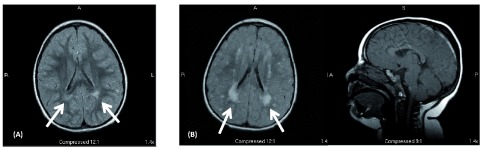
MRI findings. ( **1-A**) – Axial MRI FLAIR image from the affected female child done at 4.5 years of age shows hyperintensities in the periventricular (primarily periatrial) white matter (arrows). ( **1-B**) Axial FLAIR and sagittal T1 MRI images from the affected male child at 2.5 years of age. This shows bilateral periventricular (periatrial) and subcortical white matter hyperintensities consistent with dysmyelination (arrows). Brain architecture (corpus callosum, cerebellum, and cortical gyration) was normal.

MRI scan of the brain showed mild FLAIR/T2 hyperintensities in the periatrial white matter ( Figure 1A); a CT scan did not show calcifications. Skeletal x-rays did not show any abnormality of her bones. Hearing tests, renal ultrasound, and echocardiogram were all normal. High resolution karyotype and array comparative genome hybridization (CGH) were normal. Carbohydrate deficient transferrin study, lysosomal enzymes, and 7-dehydrocholesterol level were also normal. She has not had documented hypoglycemia or hyperglycemia. Psychometric testing led to the diagnosis of mild intellectual disability. Speech and occupational therapy were provided through school and she has continued to make steady developmental progress.

### Patient 2

The second patient is a 10 year old boy who was born at 36 weeks gestation weighing 5 pounds, and developed mild neonatal jaundice. The pregnancy was complicated by oligohydramnios and decreased fetal movement. He was diagnosed with hypotonia during infancy. He too achieved motor milestones at appropriate times (walked at 15 months) but speech was delayed. He had an episode of febrile status epilepticus at 17 months, and subsequent epileptic seizures without fever. Seizures have been controlled with levetiracetam (50 mg/kg/day, corresponding to 500 mg twice daily). A recent EEG showed symmetric, bilateral, frontally dominant polyspike and wave discharges, consistent with generalized epilepsy. He has a history of recurrent pulmonary infections and reactive airway disease; initial testing by CFTR gene sequencing was not conclusive and revealed only benign variants. Sweat chloride testing was abnormal on one occasion and then borderline, pancreatic elastase was normal, and a clinical diagnosis of cystic fibrosis was made. He also had recurrent otitis media that required tympanostomy tubes.

Physical examination at age 2.5 years showed small stature (5% for height and weight) microcephaly (head circumference 3 SD below mean for age), low anterior hair line, deep set eyes with mild hypotelorism, and a normal neurological exam. His phenotype was similar to his older sister, although he seemed to be more severely affected. MRI of the brain at age 2.5 years showed extensive FLAIR/T2 hyperintensities in the periventricular and subcortical white matter ( Figure 1B).

The following biochemical and genetic tests were normal: array CGH, fragile X test, TORCH titers, quantitative plasma amino acids, quantitative urine organic acids, fluorescent *in-situ* hybridization (FISH) test for Pelizaeus-Merzbacher disease, very long chain fatty acids, enzyme assays for CLN1 (PPT1 – palmitoyl-protein thioesterase 1) and CLN2 (TPP1-tripeptidyl-peptidase 1), and 7-dehydrocholesterol. He has not had documented hypoglycemia or hyperglycemia.

His features have remained constant on follow-up examinations, with persistent microcephaly and mild hypotelorism. He is making slow progress in school with speech, occupational and physical therapy.

## Ethics

Patients and parents were enrolled into a clinical research protocol sponsored by the Translational Genomics Research Institute (TGen) approved by the Western Institutional Review Board, Protocol Number 20120789. After informed consent, blood samples were collected for DNA and RNA extraction. A separate buccal swab was sent for Sanger sequencing and independent confirmation of selected variants in a clinical laboratory (GeneDx, Gaithersberg, MD).

## Exome sequencing

Libraries were prepared using the Illumina’s TruSeq DNA sample preparation kit and the TruSeq exome enrichment kit (Illumina, Inc., San Diego, CA, USA), following the manufacturer’s protocol. Sequencing was done by 100-bp paired-end sequencing on a HiSeq2000 instrument (Illumina, Inc., San Diego, CA, USA). Reads were aligned to the Human Genome (Hg19/GRC37) using Burrows-Wheeler transform alignment ( BWA v.0.7.5) ^1^. PCR duplicates were removed using Picard v.1.92 ^2^, and base quality recalibration, indel realignment and single nucleotide polymorphism (SNP) and indel discovery were performed using the Genome Analysis Toolkit ( GATK v.2.5-2) ^3^. Variants were annotated with SnpEff 3.2a and selected ( SnpSift) for protein-coding events. Prediction scores were loaded from dbNSFP and used for filtering.

## Results

Exome sequencing identified 33,640 SNPs and indels common to the two affected individuals. The majority of these variants were common in the population, and only 4,355 were considered either novel, private or rare variants. An annotated variant file was created that included variants in any of the four family members (male child, female child, father, and mother). Excluding UTR (untranslated region) and synonymous variants left 452 candidate variants.

There were no variants consistent with *de novo* mutation in both children (with presumed germline mosaicism in a parent). Four variants were identified consistent with a homozygous recessive model: *SERINC2, PTH2R, SGK223, and PMP22*. None of these were consistent with the clinical phenotype.

We considered an X-linked recessive model (female child less severe than male) and identified variants were in *NHS* (Nance-Horan syndrome), *DDX53, WAS*, and *TREX2*; four variants in *RPGR*. The *TREX2* variant has been reported in 83 hemizygotes in the ExAC Browser database v 0.3 (Broad Institute). The *DDX53* variant is rare and has been implicated as a potential X-linked autism locus. X chromosome inactivation studies in the carrier mother have not been done.

When we considered compound heterozygous model, we identified only two variants in a single gene, *TRMT10A*. Both children have inherited a maternal variant encoding a nonsense mutation, c.277C>T (p.Arg93*) and a paternal variant encoding a nonsense mutation, c.397C>T (p.Arg133*). The Arg133* variant has been observed in one of 120,000 alleles in the ExAC Browser database, while the Arg93* allele has never been reported before. Based on the damaging nature of these mutations (protein truncation), consistency with an autosomal recessive model, and two previous reports of homozygous *TRMT10A* mutations in children with primary microcephaly ^4, 5^, we concluded that the *TRMT10A* mutations were causal for our patients’ phenotype.

## Discussion

Microcephaly is a clinical condition, rather than a specific diagnosis, defined as having a small brain size, measured by head circumference. It is generally accepted that head circumference less than 2–4 standard deviations below the mean for age and gender is considered to be microcephaly ^6, 7^.

Primary microcephaly is either congenital and present at birth, or progressive and caused by postnatal decrease in head circumference growth rate (as in Rett syndrome, for instance). Congenital microcephaly occurs more frequently in patients born to consanguineous parents. Congenital microcephaly can be further classified by clinical and imaging features set forth by Barkovich and others ^8, 9^. Patients with autosomal recessive primary microcephaly (MCPH) have microcephaly at birth and non-progressive mental retardation ^10, 11^. Microcephaly with normal or simplified gyration (MSG) is thought to be a result of abnormal neuronal and glial proliferation or apoptosis ^12^.

Many genes have been implicated in the development of microcephaly (summarized in Table 1) including *ASPM*, *MCPH1*, *CDK5RAP2*, *CEP152*, *CENPJ*, *WDR62*, *STIL*, *CASC5*, *CEP135*, *ZNF335*, *PHC1*, *CDK6*, with many of them contributing to centrosome and spindle protein dysfunction during mitosis ^6, 10, 13– 17^. Mutations in the *ASPM* gene are the most common in this group, and account for 30–40 percent of these cases ^18^. Genes implicated in primary microcephaly with associated features such as cognitive and motor impairment and epilepsy include *SLC25A19*, *ATR*, *ARFGEF2*, and *RAB3GAP1* ^19^. *PYCR2* mutation described by Nakayama causes dysfunctional proline synthesis and leaves affected patients more susceptible to oxidative stress induced apoptosis, leading to postnatal microcephaly from cell death in the developing brain ^20^.

**Table 1.** Genes causing microcephaly syndromes. This table contains a summary of selected primary microcephaly syndromes, including MCPH 1–15. Information provided includes a brief description of the phenotype, gene that is mutated, and pathogenic mechanism.

Syndrome	Phenotype	Inheritance	Gene	Chromosome	Protein function/ pathogenic mechanism
MCPH1 (OMIM#251200)	microcephaly, intellectual disability, epilepsy, craniofacial dysmorphism, growth retardation	AR	MCPH1	8p23.1	premature chromosomal condensation; defective G2/M checkpoint arrest, CDK1 phosphorylation
MCPH2 (OMIM#604317)	microcephaly, simplified gyration, cortical malformations, epilepsy, craniofacial dysmorphism, growth retardation	AR	WDR62	10q13.12	localized to spindle poles during mitosis
MCPH3 (OMIM#604804)	microcephaly, intellectual disability, simplified gyration, developmental and psychomotor delay, cortical malformations, craniofacial dysmorphism, hypotonia	AR	CDK5RAP2	9q33.2	centrosomal function and spindle orientation
MCPH4 (OMIM#604321)	microcephaly, intellectual disability, growth retardation	AR	CASC5	15q15.1	kinetochore protein, centromere attachment to microtubules, and spindle- assembly checkpoint signaling
MCPH5 (OMIM#6-8716)	microcephaly, simplified gyration, cortical malformations, intellectual disability	AR	ASPM	1q31.3	abnormal spindle gene; mitotic spindle function in neuroblasts, cleavage plane alteration, asymmetric division
MCPH6 (OMIM#608393)	microcephaly, severe intellectual disability, craniofacial dysmorphism, epilepsy, joint stiffness	AR	CENPJ	13q12.12	centromeric protein J, microtubule assembly and nucleation
MCPH7 (OMIM#612703)	microcephaly, intellectual disability, short stature, strabismus, ataxia, epilepsy, T cell ALL	AR	STIL	1p33	neuronal cell proliferation; nuclear localization signal of TGF beta
MCPH8 (OMIM#614673)	microcephaly, craniofacial dysmorphism	AR	CEP135	4q12	fragmented centrosomes, disorganized microtubules
MCPH9 (OMIM#614852)	microcephaly, simplified gyration, psychomotor delay, jerky movements, mirror movements	AR	CEP152	15q21.1	centrosomal core protein
MCPH10 (OMIM#615095)	microcephaly, simplified gyration, cortical malformation, delayed myelination, craniofacial dysmorphism, hypertonia, spasticity	AR	ZNF335	20q13.12	zinc finger protein; neurogenesis, neuronal differentiation
MCPH11 (OMIM#615414)	microcephaly, intellectual disability, short stature	AR	PHC1	12p13.31	polycomb repressor complex; ubiquitination, proteosome-mediated degradation, chromatin remodeling
MCPH12 (OMIM#616080)	microcephaly, intellectual disability, simplified gyral pattern, craniofacial dysmorphism	AR	CDK6	7q21.2	cyclin dependent kinase, cell cycle regulation; microtubule orientation and organization
MCPH13 (OMIM#616051)	microcephaly, simplified gyration, IUGR, craniofacial dysmorphism, short stature, psychomotor delay, absent speech, spasticity, osteopenia	AR	CENPE	4q24	kinetochore associated motor protein
MCPH14 (OMIM#616402)	microcephaly, severe intellectual disability, speech delay, aggressive behavior, epilepsy, cortical malformation	AR	SASS6	1p21.2	centrosome duplication, spindle assembly; impaired centriole formation
MCPH15 (OMIM#616486)	microcephaly, severe intellectual disability, psychomotor delay, spastic quadriparesis, hyperreflexia, hypotonia, epilepsy, cortical malformations, early death	AR	MFSD2A	1p34.2	uptake of long chain fatty acids
Mental retardation, AD 32 (OMIM#616268)	microcephaly, developmental delay, craniofacial dysmorphism, cardiac defects, ocular anomalies	AD	KAT6A	8p11.21	histone acetyltransferse; dysregulation of chromatin modification
Mental retardation, AR 5 (OMIM#611091)	microcephaly, severe intellectual disability, craniofacial dysmorphism, short stature, hypertonia	AR	NSUN2	5q15.31	trna methyltransferase; spindle assembly during mitosis and chromosome segregation
Hypomyelinating leukodystrophy 10 (OMIM#616420)	microcephaly, hypomyelination, severe motor and speech delay, craniofacial dysmorphism, hypertonia	AR	PYCR2	1q42.12	pyrroline-5-carboxylate reductase; dysfunctional proline biosynthesis, oxidative stress-induced apoptosis
TAR (thrombocytopenia absent radius) syndrome (OMIM#274000)	severe microcephaly, defective neurogenesis, thrombocytopenia- absent radius	AR	RBM8A	1q21.1	exon-junction complex; rna metabolism in cortical development, neuronal apoptosis
Primary ciliary dyskinesia 31 (OMIM#616369)	microcephaly, gestational demise, craniofacial dysmorphism, renal abnormality	AR	CENPF	1q41	kinetochore and spindle; microtubule regulation, ciliogenesis
Microcephaly and chorioretinopathy - 2 (OMIM#616171)	microcephaly, severe intellectual disability, dwarfism, retinopathy	AR	PLK4	4q28.2	Polo-like kinase, regulator of centriole biogenesis
Wolcott-Rallison syndrome (OMIM#226980)	microcephaly, simplified gyration, infantile diabetes	AR	EIF2AK3	2p11.2	serine/threonine kinase; unfolded protein response, ER stress-induced apoptosis
Microcephaly, epilepsy, diabetes-MEDS (OMIM#614231)	microcephaly, simplified gyration, epilepsy, infantile diabetes	AR	IER3IP1	18q21.1	Immediate early response interacting protein; unfolded protein response, ER stress- induced apoptosis
microcephaly, short stature, impaired glucose metabolism-MSSGM (OMIM#616033)	microcephaly, intellectual disability, short stature, adolescent onset diabetes	AR	TRMT10A	4q23	trna methyltransferase; ER stress-induced apopotosis

Primary microcephaly is a result of a defect in the generation of the appropriate number of neurons during early neural development. Cell division of neural progenitors in the neuroepithelium can be symmetric (generating two progenitors) or asymmetric (generating one progenitor and one post-mitotic neuron). Microcephaly has been associated with microtubule, centrosome, and mitotic dysfunction. Dysfunction in the mitotic machinery may cause inappropriate asymmetric cell division during neurogenesis in the developing cortex and subventricular zone ^21, 22^. Abnormal control of apoptosis during the neurogenesis phase of development can also lead to microcephaly. Studies in mice have demonstrated microcephaly due to an increase in neuronal apoptosis and depletion of the neural progenitor population ^23^. Specifically, endoplasmic reticulum stress-induced apoptosis has been shown to cause microcephaly and early onset diabetes from beta cell ER stress-induced apoptosis.

## TRMT10A and microcephaly

*TRMT10A* (also known as *RG9MTD2* – RNA (guanine 9)-methyltransferase domain-containing protein 2) is homologous to a yeast tRNA methyltransferase (Trm10) and methylates tRNAs at the m ^1^G _9_ position. *TRMT10A* is expressed in several tissues (liver, kidney, spleen, lung, fat) but at especially high levels in brain and pancreatic islet cells ^4^. Mutation of another tRNA methyltransferases, NSUN2, has been associated with microcephaly, short stature, intellectual disability, and facial dysmorphism ^24, 25^.

There are two previous reports of patients with homozygous nonsense or missense mutation of *TRMT10A* resulting in microcephaly. In the first report, Igoillo-Esteve and colleagues described 3 siblings, from a large consanguineous Moroccan family, with microcephaly, intellectual disability, short stature, and early-onset diabetes ^4^. Whole exome sequencing identified a homozygous p. Arg127* nonsense mutation in the *TRMT10A* gene in the 3 affected patients; both parents and an unaffected brother were heterozygous for this mutation. Features in the oldest patient (at age 26), in addition to microcephaly and intellectual disability, included short stature, absence seizures, development of diabetes at age 22, and a variety of dysmorphic features. Brain MRI showed normal architecture and gyration. The other two children developed diabetes at age 19 and 14 respectively. The TRMT10A Arg127* mutation resulted in greatly reduced mRNA levels (presumably by nonsense-mediated decay) and absent protein levels. Loss of TRMT10A protein induced apoptosis in rat β-cells and increased stress-induced apoptosis by many endogenous molecules including high levels of glucose ^4^.

In a second report, Gillis and colleagues described 3 siblings (one female and two male) born to healthy, non-consanguineous parents from a small, inbred Jewish community of Uzbekistan ^5^. All 3 patients suffered from microcephaly, intellectual disability, short stature, seizures, and altered glucose metabolism. The female child had delayed pubertal development and was amenorrheic at age 19 years. Exome sequencing in one affected patient followed by targeted genotyping in the rest of the family (parents, two affected sibs and 9 unaffected sibs) defined a homozygous p. Gly206Arg missense mutation in *TRMT10A* as causing disease in this family. All three affected patients had episodes of hypoglycemia, hyperinsulinemia, and abnormal response to glucose. Brain imaging in two of these patients was normal. The Gly206Arg mutation resulted in almost complete loss of methyltransferase activity ^5^. This suggested that decrease in methylated tRNA is responsible for growth failure, developmental delay and impaired glucose metabolism.

TRMT10A microcephaly, in contrast to primary microcephaly linked to centrosome and spindle function during mitosis, is likely related to the loss of brain volume due to increased apoptosis. TRMT10A deficiency increases the amount of beta cell apoptosis, and presumably neuronal apoptosis. The development of diabetes and abnormal glucose response may be related to endoplasmic reticulum stress-induced apoptosis induced by endogenous fatty acids and high levels of glucose ^4^. Endoplasmic reticulum stress-induced apoptosis has also been implicated in a syndrome of primary microcephaly associated with simplified gyral pattern, epilepsy, and infantile diabetes caused by mutation of the *IER3IP1* gene ^19^.

Here we report two additional patients with primary (congenital) microcephaly and intellectual disability caused by compound heterozygous nonsense mutations of *TRMT10A*. These patients have not displayed hypoglycemia nor early onset diabetes. Brain imaging did show abnormalities in the central white matter, suggesting delayed or abnormal myelination. *TRMT10A* is not currently included in microcephaly gene panels available from commercial laboratories, stressing the importance of exome sequencing in the genetic diagnosis of primary microcephaly. The correct genetic diagnosis in our patients allows for anticipation and early treatment of medical complications by institution of meticulous glucose monitoring and control from an early age.

## Consent

Written informed consent for publication of their clinical details and clinical images was obtained from the parent of the patients.
