# Supplementary material for: Case Report: Compound heterozygous nonsense mutations in TRMT10A are associated with microcephaly, delayed development, and periventricular white matter hyperintensities
Source: F1000Res. 2015 Sep 28;4:912. [Version 1] doi: 10.12688/f1000research.7106.1 (PMC4617320; doi:10.12688/f1000research.7106.1)
Supplement: Peer review history

# Peer review: Referee response for version 1

**DOI:** 10.5256/f1000research.7652.r10547

**Published:** 2015-10-19

**Authors:** Ganeshwaran H. Mochida

**Affiliations:** 1 Pediatric Neurology Unit, Department of Neurology, Massachusetts General Hospital, Boston, MA, USA

This paper describes two siblings with microcephaly and developmental delay, who were found to have compound heterozygous variants in the TRMT10A gene. Biallelic mutations of TRMT10A have recently been reported in individuals with microcephaly and abnormalities in glucose homeostasis. Seeing that only a small number of individuals reported with this condition to date, this paper adds additional insights into this condition. The technique used in this paper is standard whole-exome sequencing. Though the manuscript does not include functional studies of the identified TRMT10A variants, these variants are predicted to lead to a premature truncation of the protein and are therefore likely to be pathogenic.

I have following comments and suggestions:

- It would be helpful to include the actual physical measurements (not just standard deviations) for the affected individuals.
- It is not explicitly stated which individuals in the family had exome sequencing done and if the TRMT10A variants were confirmed by Sanger sequencing.
- Even though there seems to be only one protein isoform for TRMT10A in the public databases, the ID of the reference sequence used for annotation of the mutations should be included.
- There are several differences in clinical manifestation between the affected individuals described in this manuscript and those previously reported. Specifically, the individuals described herein do not have obvious alteration of glucose homeostasis. Also, the cerebral white matter signal abnormalities on brain MRI have not been reported previously. These features are clinically important, and it would be helpful to discuss these points more in depth.

I have read this submission. I believe that I have an appropriate level of expertise to confirm that it is of an acceptable scientific standard.

# Peer review: Referee response for version 1

**DOI:** 10.5256/f1000research.7652.r10548

**Published:** 2015-10-13

**Authors:** Gihan Tennekoon

**Affiliations:** 1 Department of Neurology, Children's Hospital of Philadelphia, Philadelphia, PA, USA

TRMT10A is an tRNA (guanine) methyltransferase that is highly expressed in the pancreatic islet cells and brain. In the brain it appears expression occurs early during gestation and expression is in the dorsal telencephalon with high expression in the ventricular zone. Later there is expression in the cerebellum. 

This case report describes 2 siblings where the male is more severely affected. From previous reports and these 2 cases it appears to be an autosomal recessive disorder that is characterized by mild dysmorphic features including short stature, microcephaly, non-progressive intellectual disability predominantly affecting speech, no gross for fine motor difficulties and no cerebellar dysfunction. Previous reports have not described any specific changes on the imaging studies whereas this report shows periatrial and periventricular white matter changes consistent with delayed myelination. Furthermore, neither of these children had difficulties with glucose metabolism. In term of understanding the pathophysiology of how TRMT10A brain development there is more work needed.

Nevertheless, in the setting of primary microcephaly with speech delay and abnormal glucose homeostasis, TRMT10A mutations should be considered.

I have read this submission. I believe that I have an appropriate level of expertise to confirm that it is of an acceptable scientific standard.
